# Structural Features of the Interaction between Human 8-Oxoguanine DNA Glycosylase hOGG1 and DNA

**Published:** 2014

**Authors:** V. V. Koval, D. G. Knorre, O. S. Fedorova

**Affiliations:** Institute of Chemical Biology and Fundamental Medicine, Siberian Branch of the Russian Academy of Sciences, Lavrentyev Ave., 8, Novosibirsk, 630090, Russia; Novosibirsk State University, Pirogova Str., 2, Novosibirsk, 630090, Russia

**Keywords:** protein-nucleic acid recognition, human 8-oxoguanine DNA glycosylase, repair enzymes, loss-offunction mutants, structural analysis of hOGG1

## Abstract

The purpose of the present review is to summarize the data related with the structural features of interaction between the human repair enzyme 8-oxoguanine DNA glycosylase (hOGG1) and DNA. The review covers the questions concerning the role of individual amino acids of hOGG1 in the specific recognition of the oxidized DNA bases, formation of the enzyme–substrate complex, and excision of the lesion bases from DNA. Attention is also focused upon conformational changes in the enzyme active site and disruption of enzyme activity as a result of amino acid mutations. The mechanism of damaged bases release from DNA induced by hOGG1 is discussed in the context of structural dynamics.

## INTRODUCTION

The vast majority of the tertiary structures of proteins and their complexes elucidated and deposited in databases over the past half-century (as of April 2014, there are about 100,000 entries in the archive for the three-dimensional structural data, the Protein Data Bank, or PDB) have naturally turned the attention to the relationship between proteins' structures and their functions. This paper reviews the structural features of hOGG1 enzyme interaction with DNA damage sites.

The genomes of all living organisms are continuously exposed to a heavy load of exogenous and endogenous mutagens, some of which are reactive oxygen species (ROS), highly reactive cellular by-products, xenobiotics, UV light, ionizing radiation, etc. Among the wide range of agents that attack DNA [1-4] and cause numerous diseases [5-10], an important role is attributed to the ROS, such as O2'-, H_2_O_2_, and OH' [11, 12], produced under aerobic conditions. The mutagenic lesions occurring due to oxidative damage of purine DNA residues involve the 7*,*8-dihydro-8-oxoguanine (8-oxoguanine, 8-oxoG) and 5-formamidopyrimidine derivatives of adenine, 4,6-diamino-5-formamidopyrimidine (Fapy A), and guanine, 2,6- diamino-4-oxy-5-formamidopyrimidine (Fapy G) [13, 14].

A buildup of DNA lesions causes structural damage to the DNA molecule. For example, 8-oxoguanine can mispair with adenine in the Hoogsteen mode, which results in a C to A substitution in the first replication cycle (a 8-oxoG/A mismatched pair is formed), followed by the incorporation of T opposite A in the second round of replication, thereby producing a G/C → T/A transversion [15, 16].

To prevent 8-oxoG accumulation in DNA, there is a pathway to protect cells against the mutagenic effect (the GO-system) [17, 18]. This pathway has been described in details for *Escherichia coli*. It consists of three enzymes: Fpg (MutM), a specific N-glycosylase/ AP-lyase that catalyzes the excision of 8-oxoG; MutY, a specific N-glycosylase that removes adenine opposite 8-oxoG; and MutT, a nucleotide hydrolase involved in the cleavage of the pyrophosphate bond in 8-oxodGTP. Eukaryotic cells have structural or functional analogs of these bacterial enzymes [19, 20]. The excision of 8-oxoguanine from DNA in eukaryotes is carried out by the 8-oxoguanine glycosylase (OGG1) [21]. Each human cell has been shown to contain approximately 50 thousand copies of OGG1 that protect the genomic DNA from the accumulation of oxidized purine nucleotides [22].

In human cells, the *OGG1 *gene is located on the short arm of Chromosome 3 (3p25/26). The OGG1 primary transcript gives rise to two distinct mRNAs encoding proteins of the 345 and 424 amino acids; α-hOGG1 and β-hOGG1, respectively [21, 23-6]. Both isoforms share the first 316 amino acid residues, with the C-termini varying [21, 23-7]. Studies aimed at addressing their cellular location show that α-hOGG1 localizes in the nucleus, while β-hOGG1 localizes in mitochondria [27]. The nuclear isoform of α-hOGG1 is highly conserved and is well studied in humans, the yeast *Saccharomyces cerevisiae*, the plant *Arabidopsis thaliana *cells, the fruit fly *Drosophila melanogaster cells, *and mammals [28]. The α-hOGG1 of *Saccharomyces cerevisiae *and humans share up to a 38% homology. The β-hOGG1 isoform localizes only in mitochondria [28]. Catalytic and structural characteristics have been addressed for only α-hOGG1.

## THE CATALYTIC MECHANISM OF THE OGG1 PROTEIN

The hOGG1 enzyme acts both as a DNA-glycosylase and a β–lyase, hydrolysing the N-glycosidic bond of the damaged base to release 8-oxoG, followed by catalytic cleavage of the 3'-phosphodiester bond. The chemical mechanism of action was first suggested by Wallace* et al. *for endonuclease III of *E. coli *[29]. The main ideas proposed in that study were experimentally proved by Lloyd *et al*. [30] using the analysis of cross-links formed between the enzyme and DNA molecule. According to the proposed mechanism, upon incubation of DNA and the enzyme, followed by sodium borohydride treatment, a covalent bond was formed between the two molecules evidencing the occurrence of Schiff base intermediate.

**Fig. 1:**
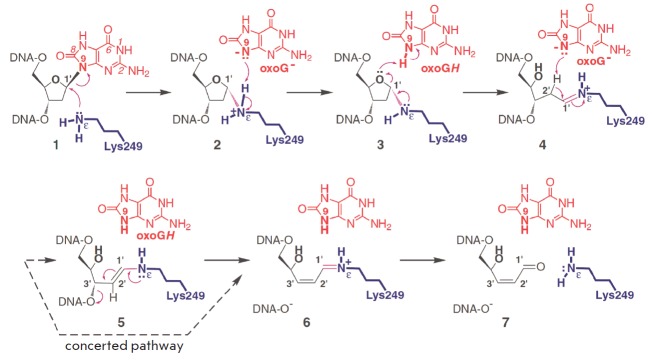
Detailed stepwise mechanistic proposal for the entire cascade of reactions catalyzed by hOGG1 in which the reaction product serves to catalyze further processing of the substrate. (Reprinted by permission from Macmillan Publishers Ltd: [Nat. Struct. Biol.] Fromme J.C., Bruner S.D., Yang W., Karplus M., Verdine G.L. Nat. Struct. Biol. 2003. V. 10. № 3. P. 204–211, copyright 2003)

The catalytic action of hOGG1 involves a mechanism by which the ε-amino group of Lys249 participates in the removal of the 8-oxoG base from the C1' of the ribose moiety and promotes the elimination of the 3'-phosphodiester bond through the formation of a Schiff base intermediate with C1' of the deoxyribose moiety (*Fig. 1*) [31-33]. Verdine *et al. *earlier showed that K249Q mutant hOGG1 exhibited no catalytic activity but retained the ability for the recognition of oxidatively damaged DNA [21]. The second chemical event is the scission of phosphodiester bond at the C3 of the 2'- deoxyribose moiety via β-elimination (AP-lyase activity).

Based on numerous structural and biochemical studies, Verdine *et al. *[34] proposed a role of 8-oxoG being displaced in acid/base interactions with the Nε, (Lys249 amino group), C2', and O4' atoms. The structure of the intermediate reduced by sodium borohydride, produced through the reaction of hOGG1 with 8-oxoG-containing DNA, has been characterized. The 8-oxoG base to be removed from the DNA strand is not released but retained in the active site and acts as a cofactor at the β-elimination stage. The N9 of the enzyme in the 8-oxoG base is positioned near Nε and O4', which permits the transfer of a proton between these two atoms.

*Fig. 1* illustrates the enzymatic mechanism of hOGG1 as described [34]. The initial attack of the sidechain amino group of Lys249 on the C1' of the deoxyribose sugar results in cleavage of the glycosidic bond to produce an 8-oxoG- anion. The anion deprotonates the ε-NH2 of Lys249 to form the aminal intermediate** 3**. The protonated 8-oxoGH transfers the proton to the O4' of the ribose sugar, with the aminal intermediate** 3 **rearranging to **a **Schiff base **4 **with a sugar ring opening*. *The Schiff base **4 **with a proton on the Nε of Lys249 donates it back to 8-oxoG- to yield 8-oxoGH and an uncharged Schiff base **5 **promoting the loss of the 3' phosphate via β-elimination. DNA with a 5' phosphate group and a positively charged intermediate **6 **arise. The intermediate carries an α,β-unsaturated Schiff base at the 3' end with a positive charge. Upon hydrolysis, the intermediate **6 **releases the enzyme and a DNA molecule with 4-hydroxy-2,3-pentenal-1 at the 3'end.

The Protein Data Bank currently holds information on 27 structures of hOGG1. The crystal structures of native hOGG1 [35] and DNA-bound hOGG1 have been solved: catalytically inactive K249Q mutant hOGG1 with 8-oxoG-containing DNA [35, 36], N149C with 8-oxoG-containing DNA and intact DNA [37], D268N with 8-oxoG-containing DNA and DNA with a tetrahydrofuran residue (F-ligand) –a “stop” substrate for hOGG1 [33, 38]; and the WT hOGG1 complex with the F-ligand [33]. In addition, the irreversibly linked adduct of hOGG1 with AP substrate has been described [34], formed through a borohydride-trapped Schiff bases for hOGG1 variant forms (H270A, Q315A, Q315F, G42A) containing mutation of amino acids that bind 7*,*8-dihydro-8-oxoguanine in the native structure [32]. The structural analysis has also been conducted for a late-stage intermediate wherein 8-oxoG is almost completely inserted in the active site; however, catalytically active conformation has not been achieved yet [32].

## STRUCTURAL CHARACTERISTICS OF K249Q hOGG1 LACKING ENZYMATIC ACTIVITY

**Fig. 2:**
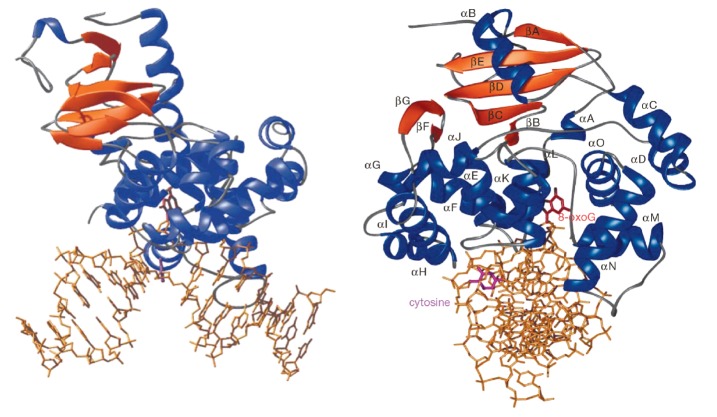
The overall structure of the hOGG1-DNA complex. Two orthogonal views of the hOGG1-DNA complex with the protein shown as ribbon (blue, α-helices; orange, β-sheets; gray, elements with no secondary structure) and 15-base pair DNA oligonucleotide, as sticks (gold). The substrate oxoG base (red) is completely extruded from the DNA helix and inserted into an active site pocket. The complementary or “estranged” cytosine (purple) remains stacked in the helix. The enzyme bends the DNA (~70°) at the plane of the oxoG·C base pair. The bend in the DNA exposes the edge of the estranged cytosine to protein side-chains that make specific contacts. (Reprinted by permission from Macmillan Publishers Ltd: [Nature] Bruner S.D., Norman D.P.G., Verdine G.L. (2000) Nature. 403. 859–866, copyright 2000)

The first structure of complex of hOGG1 with 8-oxoG-containing substrate was obtained for hOGG1 with a K149Q mutation [36]. It was shown earlier [21] that the mutant form, in which Lys249 is replaced with Glu, lacks catalytic activity but retains substrate recognition. Since the authors [36] failed to yield high-quality crystals for full-length hOGG1 K249Q complexed with the DNA duplex containing 8-oxoG/C, limited digestion by trypsin allowed to remove unstructured aminoand carboxyl-termini as well as amino acids at positions 80–2, facilitating crystallization and the analysis of the hOGG1 core domain comprising residues 12–25 (*Fig. 2*).

It has been demonstrated that hOGG1 shares a common fold with the members of the superfamily of DNA repair enzymes involved in base excision repair (BER), such as endonuclease III and alkyl-DNA-glycosylase AlkA from *E. coli *[39]. The repair proteins of this family occur in numerous organisms, from bacteria to mammals, repairing DNA bases damaged by oxidation*,* alkylation, and deamination. These enzymes possess a unique structural motif “helix-hairpin-helix” (HhH) [40], followed by a Gly/Pro-rich loop and conserved residues of Gly, Pro and Asp (HhH-GPD). The hOGG1 structure also contains two α-helix domains shared by all members of the HhH-GPD superfamily and antiparallel β-sheet present in the alkyl-DNA-glycosylase AlkA only.

The protein has high affinity to 8-oxoG-containing DNA (*Fig. 2*). The 8-oxoG base flips out of the DNA helix and fits into the pocket of the enzyme's active site, which is in agreement with similar structures in other members of the HhH-GPD superfamily [31, 40, 41]. In the case of the 8-oxo-dG base, the heterocyclic compound exists in the *syn*-conformation in relation to the glycosidic bond; although, upon binding to the active site of hOGG1, it takes the *anti*-conformation; i.e., as is the case for a normal duplex DNA. The flipped-out conformation of the glycosilic moiety and DNA backbone leads to the extrusion of 8-oxoG out of the DNA helix and insertion deeply into the active site of hOGG1.

**Fig. 3:**
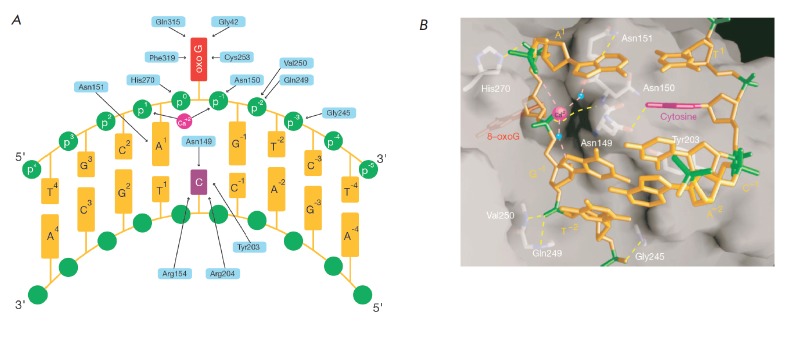
Contact interface between hOGG1 and oxoG·C-containing DNA. A – The DNA-protein interface. Arrows indicate interactions between amino-acid residues (blue) and DNA bases (yellow). B – Surface of the DNA-protein interface. Hydrogen bonds are shown in yellow, and coordinative interactions with a calcium ion (magenta) are shown in pink. (Reprinted by permission from Macmillan Publishers Ltd: [Nature] Bruner S.D., Norman D.P.G., Verdine G.L. (2000) Nature. 403. 859–866, copyright 2000)

The interaction of hOGG1 with the phosphate groups of 8-oxoG-containing DNA, 8-oxoG itself, and the complementary cytosine contribute to a contact surface of 2.268 A^2^ [36]. Though in most DNA-binding proteins the contact region contains many lysine and arginine residues, for the interaction with the phosphates groups of hOGG1, the DNA backbone is bound to a nearly uncharged groove lined by a single basic His270 residue. The resulting complex is unique in a way that hOGG1 contains many α-helices with the N-termini oriented towards the DNA (*Fig. 2*). This disposition of α-helices enhances the helix-dipole interaction, promoting dipole electrostatic contacts rather than salt bridges while binding DNA substrates. Only one of the helices, αL, is involved in direct contact with the DNA backbone. The αL helix with a loop and the αK helix form the conserved motif HhH. In addition to the interaction through the αL helix (Val250 and Gln249) and the phosphate group p^-2^, the highly conserved glycine residue (Gly245), located within the loop, forms a hydrogen bond with the phosphate p^-3^ (*Fig. 3*). The structural motif HhH is brought into con tact with a DNA substrate from the 3'-side of the oxidized base; at this site, the structure of duplex DNA is very similar to the B-form. Consequently, the HhH motif is mainly responsible for positioning the mutated base in the DNA duplex towards the active site pocket.

The phosphate groups p^-1^, p^0^ and p^1^ play an important role in the stabilization of the uncommon DNA backbone conformation at the mutagenic site. The rotation required for flipping 8-oxoG out of the helix causes inward rotation of oxygen atoms of p^-1^ and imposes extra strain on the ribose phosphate DNA backbone. To decrease electrostatic repulsion between the closely positioned p^-1^ and p^1^, a partially hydrated Ca^2+^ ion, present in the crystal microenvironment, is brought in between and secured through a direct bond with p1 and the water bridge with p-1 (*Fig. 3B*). Although Ca^2+^ could be displaced by Mg^2+^ under physiological conditions, it does not come in direct contact with the protein, but its ligand, the water molecule, forms a hydrogen bond with DNA, thereby stabilizing its flipped-out and bent conformation.

The complementary cytosine locates inside the helix; however, it hardly stacks with neighboring nucleobases from the 5' side due to a kink in the chain that orients the duplex away from the enzyme. Beyond the active site, the DNA conformation is similar to that of canonical B-DNA form (*Fig. 2*).

The void remaining in the duplex after extrusion of 8-oxoG, is filled in by the amino acid residue of the conserved NNN-element (a motif of three Asn residues); namely, Asn149 that forms a hydrogen bond via its side-chain amide carbonyl with the exocyclic NH_2_ of the estranged cytosine (*Fig. 3B*). In addition, hOGG1 plunges the indole ring of Tyr203 into the space between C° (estranged cytosine) and the base on the 5'-side (*Fig. 3B*), thus unstacking the two bases and creating a sharp kink in the DNA molecule, which significantly improves access to the Watson-Crick edge of base from the minor-groove side. C° gets unstacked from the base on its 3'-side (*Fig. 3B* T^1^ base). The residues Arg154 and Arg204 of hOGG1 move together toward C° from the minor groove; one arginine above and the other below the plane of the pyrimidine ring, simultaneously creating hydrogen bonds with the acceptor N3 and O2 atoms of the estranged C°. These bonds seem to be exceptionally strong and occur in the presence of adjacent acceptor atoms, which are unique to cytosine among the other DNA bases. Along with the interaction of Asn149 and C° amide carbonyl of the enzyme and the estranged cytosine, respectively, up to five hydrogen bonds could be created.

The role of Asn149 has been elucidated in the work [35] that demonstrated that Asn149 formed a hydrogen bond with N4 of the exocyclic NH2 of the cytosine pairing with 8-oxoG. The hydrogen bonds made by the guanidine group of Arg204 with the N1 and O2 form a unique recognition site for the estranged cytosine and appear to play an essential role in the specific recognition of the estranged cytosine typical of hOGG1.

Recognition of 8-oxoG at the active site is accomplished by specific contacts between the lesion base and the amino acids. The enzyme recognizes the urea fragment in 8-oxoG containing the C8-carbonyl group, the N7 and N9 atoms, with N7 forming a hydrogen bond with the carbonyl of Gly42. Of all contacts associated with 8-oxoG, only the one made by Gly42 that would be different with oxoG versus guanine. Therefore, the authors of [36] conclude that 8-oxoG is discriminated from G by a single hydrogen bond. Significantly, the essential amino acid residue Gly42 is the only residue that the β-sheet domain contributes to the hOGG1–DNA interface.

**Fig. 4:**
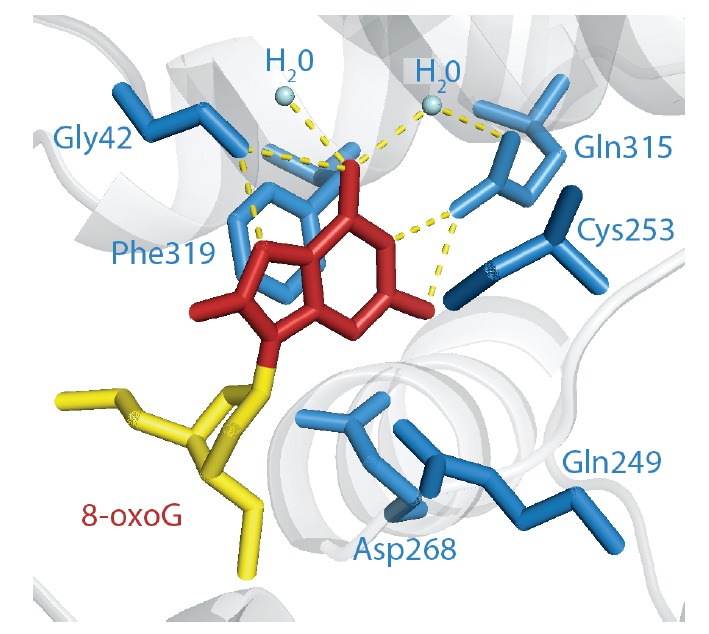
Amino acids of the hOGG1 active site involved in the recognition of the 8-oxoG base. Visualization of the 3D structure (PDB ID: 1EBM [36]) using the software package PyMOL [43]

In addition to the aforementioned residues responsible for 8-oxoG recognition, other residues of the hOGG1 active site also contribute to recognition. Phe319 and Cys253 stack toward opposite π-faces of the 8-oxoG, sandwiching the base in the active site (*Fig. 4*). The Gln315 amide NH2- group, in cooperation with a tightly bound water molecule, interacts with O6 of 8-oxoG, and the Gln315 side-chain carbonyl forms two hydrogen bonds with N1 and N2H of 8-oxoG. Another tightly bound water molecule is hydrogen bonded to O6. Gln315 and Gly42, including the water molecules trapped in the active site, are not chemically able to form hydrogen bonds with A, C, and T through donor/ acceptor interactions.

The structural analysis in [36] provides insight into the role of the catalytically important residues Lys249 and Asp268. Lys249 is located ~2.5 A off the C1' of 8-oxoG close to the space in the active site into which the lesion base is extruded, with the Asp268 is suitably disposed to assist the protonation/deprotonation of Lys249. The intermediate, formed by an attack of the deoxyribose sugar by Lys249, rearranges to a Schiff base (*Fig. 1*). This rearrangement requires deprotonation of the Lys249 side-chain amino group, presumably by Asp268, and O1' protonation, for which His270 is suitably positioned. This role of His270 sheds light on why this residue is invariant in members of the HhHGPD superfamily, since it is involved in the catalyzing of the formation of Schiff base intermediates.

## CATALYTICALLY ACTIVE hOGG1 COMPLEX WITH THE “STOP” SUBSTRATE

The structure of hOGG1 complex with DNA containing 2-oxomethyl*-*2*-*oxo-tetrahydrofuran (F) instead of 8-oxoG has been reported in [33]. This DNA substrate acts as a stop substrate for hOGG1. It is shown that in the produced structure (PDB ID: 1FN7) Asp268 is positioned rather far from Lys249 to initiate deprotonation of its side-chain amino group; the N–O distance is 3.7 A. In addition, Asp268 does not contact with the His270 that is likely needed for hydrogen bonding to O1' of the ribose sugar as it was found in K249Q hOGG1 structure [36]. Taken together, the absence of the lesion base being flipped out of the helix and into the active site pocket causes conformational changes in the enzyme.

Based on the findings of [33], it was concluded that, firstly, the 8-oxoG-recognizing pocket in hOGG1 well fits the structure of the lesion, positioning Phe319, His270, and Asp268 in the appropriate arrangement. Secondly, the role of Asp268 in the deprotonating of Lys249 has been neither confirmed nor excluded. A hypothesis is proposed that this residue creates an electrostatic field that stabilizes the positive charge developing in the transition state, especially at O1' and C1' of the 8-oxoG deoxyribose.

## hOGG1 CONFORMATIONAL CHANGES UPON DNA BINDING

**Fig. 5:**
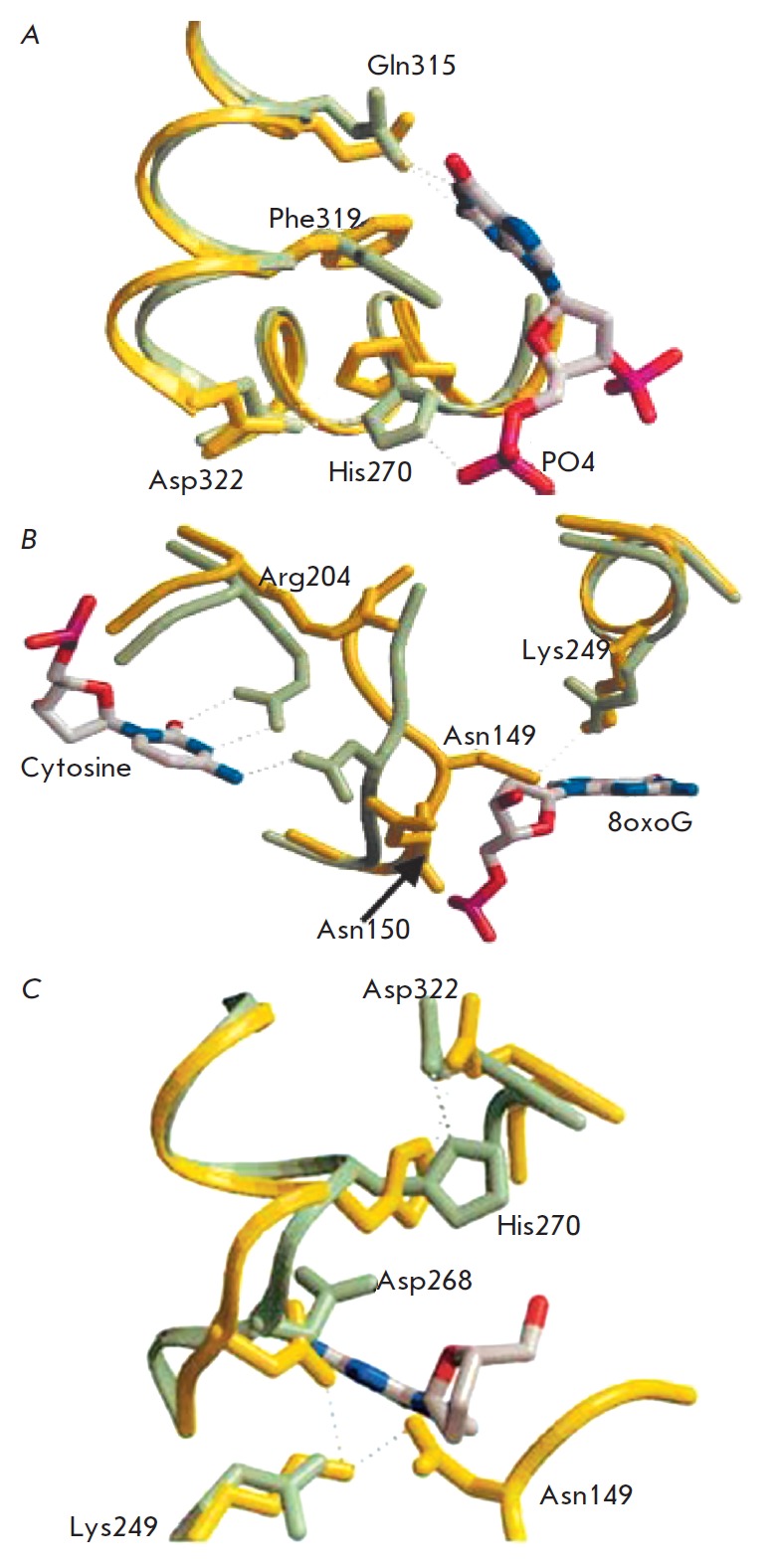
Recognition of the 8-oxoG base by hOGG1 (the free enzyme is shown in yellow; hOGG1 in complex with DNA is shown in green). A – The conformation of His270 in native and DNA-bound structures together with interactions involving Gln315, Phe319, and Asp322 which form a trigger that switches between the closed and open states of the 8-oxoG binding pocket. B – Involvement of Asn149 in the recognition and binding of complementary cytosine. C – Conformational changes in the localization of Asp268 and His270 during binding of B 8-oxoG. (Reprinted by permission from Elsevier Science Ltd.: [J. Mol. Biol.] Bjørås M., Seeberg E., Luna L., Pearl L.H., Barrett T.E. J. Mol. Biol. 2002. 317. 171–177, copyright 2002)

The crystal structures of native hOGG1 and that bound to 8-oxoG-containing DNA were obtained and described by Bjoras *et al.* [35] at a resolution of 2.15 A. It was found that the hOGG1 structure significantly differs from those of the free form and the one complexed with 8-oxoG-containing DNA (*Fig. 5*). The Phe319, Cys253, Gly42, Gln43, Phe45, and Gln315 residues are responsible for recognition, which is consistent with other findings [36]. Phe319 and Cys253 sandwich 8-oxoG, whereas Gly42, Gln43, and Phe45 interact with the major-groove edge, recognizing the protonated N7 of 8-oxoG. The Gln315 amide oxygen is hydrogen- bonded to the N1-imino- and N2-amino groups of the ring involved in base pairing. The Phe319 residue takes different conformations in the free form of hOGG1 and that bound to DNA (*Fig. 5A*). When bound to DNA, the aromatic ring of Phe319 is oriented almost perpendicular to its position in the free form. The sidechain of Gln315 in the free enzyme is positioned under the aromatic ring of Phe319 (*Fig. 5A*). In complex with DNA, the nitrogen atom of the amide moiety of Gln315 and the carbonyl oxygen of Pro266 located on the opposite side of the binding site are involved in hydrogen bonding. This causes a significant conformational change in the enzyme, thereby creating a tight pocket for binding the damaged base.

In comparison with [36] a slightly different point of view for the role of His270 has been proposed [35]. It was demonstrated that conformational changes in the lesion-recognition pocket are accompanied by a change in the orientation of His270, which forms two hydrogen bonds when bound to DNA: one between the Nε2 of the imidazole ring and the 5'-phosphate of 8-oxoG, the other between Nδ1 and the carboxyl group of Asp322 (*Fig. 5A*). In the free enzyme, one hydrogen bond with Asp322 is retained, although the imidazole ring of His270 rotates by more than 90° as compared to the DNA-bound conformation; packs against the phenyl ring of Phe319 and forms two layers of a sandwich completed by Gln315. The His270 rotamer is not compatible with the Phe319 and Gln315 conformations required for specific binding of the 8-oxoG in DNA. The side-chain conformations of Phe319 and Gln315 depend on the His270 conformation, which is itself governed in the 8-oxoG-DNA complex by interaction with the 5'-phosphate of 8-oxoG nucleotide. Overall, binding of the ribose-phosphate backbone to DNA influences the conformation of His270, which in turn causes a conformational alteration to Phe319 and Gln315, thus allowing entry of the aberrant base into the pocket.

Hence, the side-chains in Phe319, Gln315, and His270 behave as an entity, flipping between the closed and open states when binding the 8-oxoG-base.

In the free form, the amino acid region of 146 to 151 adopts a conformation different from that in a DNAbound state wherein the atoms could shift away from their positions by up to 4–9 A. Most pronounced changes occur within the center of the motif, with the sidechain of Asn149 in the free form extending back toward the enzyme and hydrogen bonding to the amide oxygen and e-amino group of the catalytic residue Lys249 (*Fig. 5B*). However, during complex formation with 8-oxoG-DNA, this oxygen atom retracts by ≈ 9 A to pack against the estranged cytosine. The remaining residues of Asn in the NNN triplet, 150 and 151, whose hydrogen bonds in a DNA-bound structure stabilize the cytosine-recognizing protein site, are exposed to a solvent in the free form. The conformation of this inter- helical peptide seen in the free enzyme is unable to bind to 8-oxoG in its the extrahelical conformation. Flipping of this enzyme segment into target DNA, as well as flipping of the scissile base into the enzyme pocket, requires conformational alterations of the protein to allow the chemistry to take place.

An important sequel of His270 re-orientation in the hOGG1-8-oxoG-DNA complex is an incremental shift of the hinge and strand between Pro266 and Trp272, which moves the side-chain of Asp268 by over 1.5 A as compared to its position in the free enzyme (*Fig. 5C*).

In the DNA-bound hOGG1 complex with F-ligand [33], the Lys249 residue is located away from the carboxyl group of Asp268, which challenges its role in the deprotonating of the nucleophile, thus indicating an alternative function for this residue in the transition state charge stabilization of the deoxyribose ring. In addition, the carboxyl group of Asp268 and the imidazole group of His270 in the DNA-bound structure are sterically close for weak hydrogen bonding which would favor proton abstraction by Asp268, promoting its indirect catalytic contribution. On the other hand, the side-chains of Asp268 and His270 in the free hOGG1 are positioned >4 A apart, which essentialy prevents their hydrogen bonding (*Fig. 5C*). By contrast, the side-chains of Lys249 and Asp268 sterically allow a hydrogen bond formation between the ε-amino- and carboxyl groups, respectively, as well as another hydrogen bond between the ε-amino group of Lys249 and the side-chain oxygen of Asn149 (*Fig. 5C*). The side-chain configuration of Lys249 in the active site corresponds to the protonated ε-aminogroup stabilized by the neutral hydrogen bond with the amide oxygen atom of Asn149 and the hydrogen-bonded deprotonated side-chain carboxyl of Asp268.

According to the catalytic mechanism for OGG1 [29-31], Lys249 provides a nucleophile attack on the C1' of the deoxyribose moiety, displacing 8-oxoG and generating Schiff's base covalent intermediate [39]. The protonated side-chain of Lys249 in the free enzyme lacks this function that would require a lone pair of electrons as in the neutral ε-NH_2_. Therefore, the Lys249 residue should be deprotonated for the reaction to proceed. This would only be possible if the interaction with Asp268 stabilizing the protonated Lys249 is cleared upon binding to the substrate.

It was shown in [35] that binding of 8-oxo-dG promotes a change in the position of Asp268 (through movements in Phe319 and Phe270), which breaks its ion-pair/hydrogen bond to Lys249. At the same time, to promote the interaction between the enzyme and the estranged cytosine, the inter-helical hinge is extruded, accompanied by a loss of hydrogen bonding between Lys249 and the side-chain carbonyl group of Asn149. Following the removal of these neutralizing interactions, the protonated ε-amino group of Lys249 should be disfavored with regard to the neutral state generated by proton abstraction from the carboxyl group of Asp268, in parallel to its movement and loss of the hydrogen bond with Lys249.

Overall, the findings suggest [35] that the hydrogen bonds established by Asp268 and Asn149 with a protonated nitrogen of the ε-amino group of Lys249 act as trigger locks in the free enzyme. One hydrogen bond is involved in 8-oxoG recognition; the other, in the recognition of cytosine. Both should be removed for the enzyme to trigger a nucleophilic attack on the C1' of the deoxyribose.

## THE ROLE OF Asp268 IN THE CATALYTIC ACTION OF hOGG1

Asp268 is catalytically important for both hOGG1 and other members of the structural family to which it belongs [26, 44]. Substitution of residue 268 for Ala or Asn abrogates the glycosylase and AP-lyase activities; however, substrate recognition is retained.

Initially, it was suggested that Asp268 could be important for oxidized base excision through deprotonation of Lys249, thereby converting the catalytically inactive cation into a nucleophilic neutral amine [36]. However, this is inconsistent with the fact that Asp located at the end of the α-protein helix needs to be less basic by several orders of magnitude. The fixed position should prevent the rotation of Asp268 around C^α^–C^β^ to interact with Lys249. The X-ray crystal structure of the native enzyme bound to AP-DNA [33] showed that Asp268 and Lys249 have no contact. Indeed, only the free form of the protein permits hydrogen bonding between the two, with Asp268 preserving its position in the α-helix, whereas Lys249 swivels about to enable contact [35]. Importantly, even though in the case of weakly hydrogen-bonded Glu268 and Lys249 (in mutant D268E) and a longer side-chain, the side-chain of Glu268 lacks contact with Lys249. It is likely that if the p*K*_a_ of Lys249 declines slightly due to the sequence context to produce a certain amount of neutral amine, this could be sufficient to break the glycosidic bond and excise the aberrant base.

**Fig. 6:**
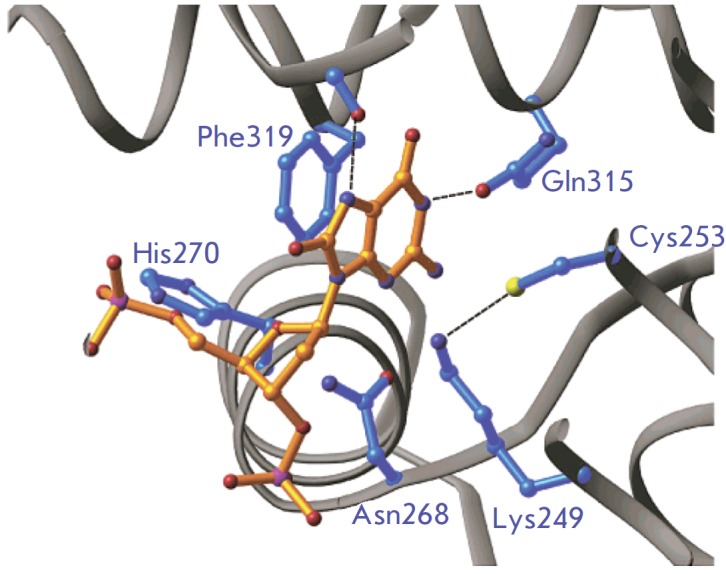
Active-site structure of D268N hOGG1 in a complex with 8-oxoG-containing DNA. Visualization of PDB ID: 1N3C [38] (Reprinted by permission from American Chemical Society: [Biochemistry] Norman D.P.G., Chung S.J., Verdine G.L. Biochemistry. 2003. 42. 1564–1572, copyright 2003)

To elucidate the role of Asp268 in the hOGG1-dependent catalysis, this residue was substituted for asparagine (D268N), glutamate (D268E), and glutamine (D268Q) [38]. It was demonstrated that Asp268, located at the N- terminus of the α-helix (*Fig. 6*), plays a dual role in the catalysis of base excision and DNA strand scission. The mutation of this residue to asparagine led to a significant decline in the enzyme activity of D268N hOGG1. The D268N crystal structure first revealed the contribution of this nucleophilic Lys residue (Lys249) in the recognition of an aberrant guanine (8-oxoG or F).

Analysis of this structure allowed to suggest that 8-oxoG excision was performed through dissociative mechanism consisting of cleavage of the glycosidic bond, deprotonation of Lys249 (perhaps, by 8-oxoG anion) followed by subsequent linkage of Lys249, forming a Schiff base between the ε-NH_2_-group and C1' of the deoxyribose sugar. On the other hand, a change from aspartic acid to glutamine (D268Q hOGG1) and glutamate (D268E hOGG1), although it led to a protein fold disruption but did not abrogate catalytic activity.

It is shown [38] that complexes of Asp268 mutated hOGG1 with DNA are structurally similar to that of K249Q mutant hOGG1 [36]. The root mean square deviation of the protein backbone coordinates between the D268N and K249Q hOGG1 structures is 0.32 A, which confidently lends credence to the identity of the structures. Comparison between the structure of wildtype and D268E mutant hOGG1 bound to F-analog yields root mean square deviations of 0.45 A and 0.59 A for WT hOGG1/F and hOGG1 D268Q/F structures, respectively. This is indicative of the fact that no global conformational changes occur following mutations of active site amino acid. In all three structures, considerable alterations were only observed in the active site, especially in the first three residues of helix α-M (residues 269, 270 and 271). In all three structures, the nucleophilic ε-NH2-group of Lys249 has a relatively fixed position, even though it is located at the end of a long alkyl chain. In this conformation, the ε-NH_2_-group of Lys249 is located near the C1' of the deoxyribose moiety (3.4 A), but in an orientation unfavorable to a straightforward nucleophilic attack on the glycosidic bond (*Fig. 6*). The ε-NH_2_-group of Lys249 seems to be hydrogen-bonded to the sulfhydryl of Cys253, which is, by contrast, attached to the π-system of the 8-oxoG through a van der Waals interaction. Since Cys253 is located on the top face of the ribose moiety, Lys249 cannot form contact with Cys253 when the sugar is attacked from the underside. In the wild-type enzyme, the carbonyl group of Asn268 is hydrogen-bonded to the His270 side-chain, whereas in the D268N mutant, the NH_2_-amide group of Asn268 is positioned too far off His270 for hydrogen bonding to occur. The absence of bonding clarifies the relatively weak binding of DNA to the D268N mutant variant, since His270 is in direct contact with the phosphate backbone on the 5'-side of the lesion. The remaining part of the active site and recognition pocket in D268N and K249Q mutant hOGG1 bound to 8-oxoG-DNA are structurally identical.

An alternative view of the role of Asp268 is the electrostatic stabilization of the positive charge on the O4' of the deoxyribose sugar in the transition state during base excision [38]. This is in agreement with the fact that in all hOGG1 structures with Asp at position 268, the carboxyl oxygen is positioned near the O4' of the sugar phosphate backbone DNA, at a distance of 3.2 A. Due to the strong interaction with its α-helix, the orientation of the Asn268 side-chain in D268N is nearly the same as that of Asp268 in the wild-type enzyme. Consequently, a change of Asp to Asn at position 268 leads to the substitution of a negatively charged oxygen for a neutral NH2 group, maintaining the position in the active site. The imide nitrogen of Asn268 and O4' of the deoxyribose moiety (3.4 A) are positioned at a longer distance than would be expected the hydrogen bonding distance between these atoms; however, this is sufficient for O4' to experience the partially positive electrostatic field of the amide protons on Asn268. It is concluded [38] that the charge change from Asp (δ^-^) to Asn (δ^+^) at position 268 increases the transition state energy for base excision due to the enchanced positive charge on O4', which results in a significant decline in reaction rates.

Taken together, it was obtained [38] that hOGG1 containing Asn in place of Asp268 (D268N) exhibits no catalytic activity. The amino acid mutations D268Q or D268E, even though they confer a sterically disfavored conformation, do not diminish the catalytic activity. These findings argue against the role of Asp268 as an acid/basic catalyst in hOGG1 [36]; however, they support its involvement in the charge appearance on the O4' of the deoxyribose sugar in 8-oxoG.

## RECOGNITION OF 8-oxoG

8-oxoG and the G base structurally differ by two positions: C8 carries O or H; and N7 contains H or a lone pair of electrons, respectively. For this reason, the H atom adjacent to N7 in 8-oxoG is capable of hydrogen bonding with the carboxyl of the Gly42 main chain, whereas G is not. To clarify the structural features of hOGG1 in complex with 8-oxoG and intact G, N149C mutant hOGG1, lacking catalytic activity, was probed tested [37]. Cys149 was connected with a linker to the C4 of cytosine complementary to 8-oxoG through a disulfide bond, thereby preventing the dissociation of the enzyme-DNA complex. The X-ray structures for N149C hOGG1 complexed with 8-oxoG-, G-, and 7-deaza-G-containing DNA duplexes have been solved.

**Fig. 7:**
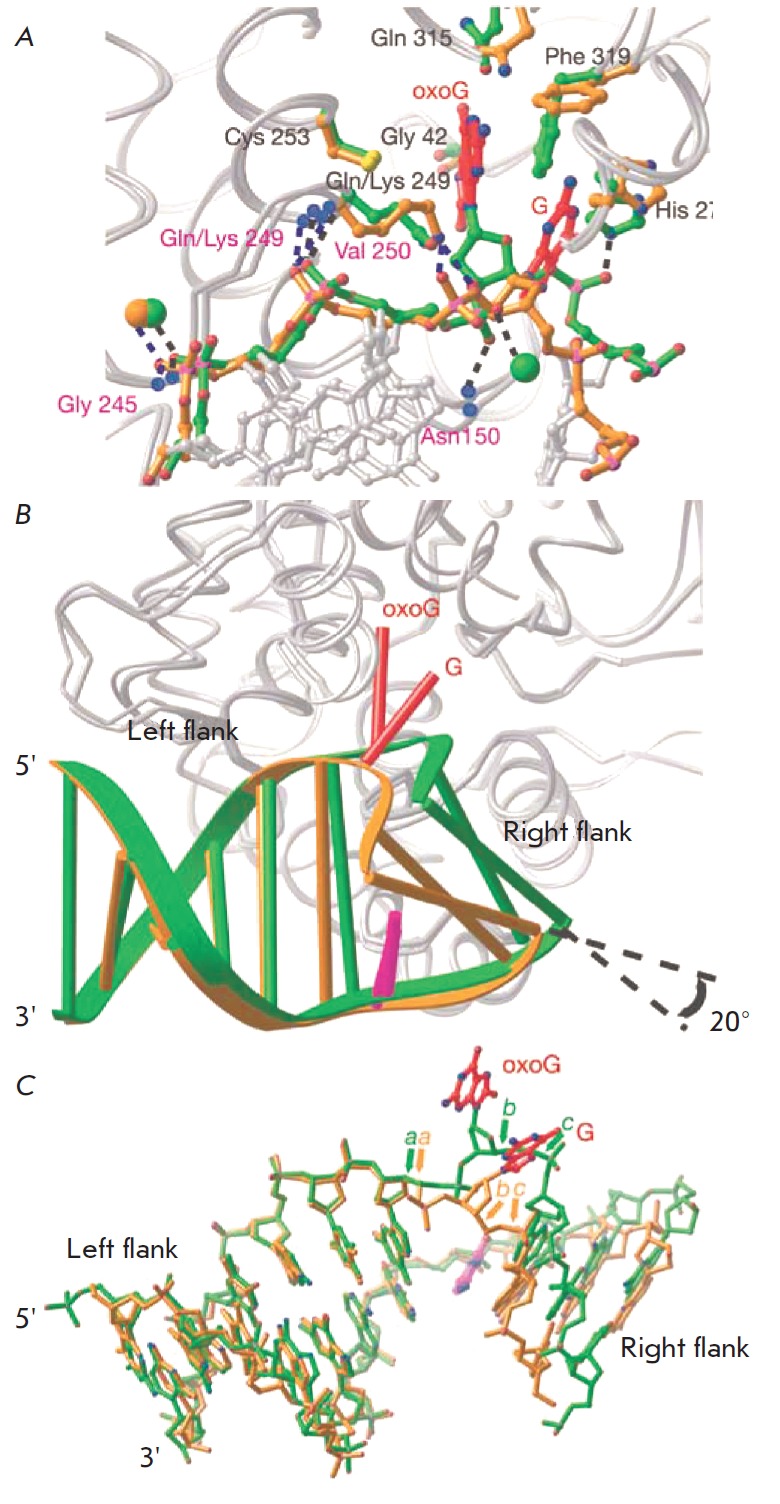
Superposition of the N149C hOGG1 complexes with 8-oxoG-containing DNA (shown in green) and with the G-containing DNA (shown in yellow) in the region around the protein–DNA interface. A – Active site residues localization during interaction with the 8-oxoG and G bases. B – Changes in the structure of the DNA duplex depending on binding of damaged or undamaged bases. C – Comparison of the DNA in the two complexes, using the left flank for superposition. Arrows labeled a, b, and c indicate bonds that have undergone significant rotations: +110° for a (C4'–C5' bond of the residue 3' to oxoG/G), +119° for b (C4'–C5' bond of oxoG/G), and –151° for c (P–O5' bond of oxoG- /G). (Reprinted by permission from Macmillan Publishers Ltd: [Nature] Banerjee A., Yang W., Karplus M., Verdine G.L. Nature. 2005. 434. 612–618, copyright 2005)

The global structures of hOGG1 enzyme complexed with 8-oxoG- and G-containing DNA reveal a drastic helical axis kinks of ~70 and ~80o , respectively, at the plane of the extruded base. The 8-oxoG base is located deeply in the active site pocket, whereas G lies against the enzyme surface at an exo-site positioned about 5 A away from the pocket (*Fig. 7 A,B*). The G base interacts with two active site residues Phe319 and His270 but these contacts differ from those made by 8-oxoG. In the 8-oxoG-containing complex, His270 is not in contact with the damaged base but forms hydrogen bond with its 5'-phosphate. In a G-containing DNA, His270 interacts with the π-system of the base, without hydrogen bonding to its phosphate.

Free energy calculations made for the interactions of 8-oxoG and G with the hOGG1 active site (ΔA_1_ and ΔA_2_ respectively) using quantum mechanics/molecular dynamics simulation techniques [37] showed that the value for free energy discrimination ΔΔA = ΔA_1_ – ΔA_2_ is -6.8 kcal/mol, which indicates a 105-fold preference for binding 8-oxoG in relation to G into the active site.

The hydrogen bond between the carbonyl oxygen of Gly42 and the proton at N7 in 8-oxoG strongly stabilizes the complex conformation. In the case of G, this will be replaced by Coulomb repulsion between the carbonyl oxygen of Gly42 and the lone pair of electrons at N7, if G and Gly42 are positioned similarly as in the 8-oxoG complex.

The study [37] provides insight into the recognition mechanism for the lesion base. It is likely that base extrusion involves more than one step and cannot occur as a single process but rather progresses through multiple discrete states. This conclusion is supported by the fact that G-bound hOGG1 could mimic the intermediate produced upon binding to 8-oxoG, immediately before flipping out into the enzyme pocket. The multi- stage recognition mechanism for an aberrant base by hOGG1 is also corroborated in the study whereby 2-aminopurine and tryptophan fluorescence emissions were used for recording the dynamics of conformation changes [45, 46].

When compared, the structures of G- and 8-oxoG-DNA bound to the enzyme reveal events taking place at the final extrusion step. At the 3'-side (*Fig. 7*, left flank) of the damaged base the structures are quite similar. They retain the hydrogen bond contact with the HhH, containing Gly245, Gln249, and Val250, as well as electrostatic interaction with the divalent metal ion (*Fig. 7A*). The only difference is the 3'-phosphate of the extra-helical nucleoside hydrogen bonded to Lys249 in the G-containing complex; whereas in the 8-oxoG-bound structure, Lys249 is unable to approach the 3'-phosphate and must rotate to face the active site to promote catalysis. It has been suggested [37] that the contacts on the 3'-side of the lesion, even more likely that of the 3' phosphate of the DNA molecule with Lys249, are made before the base extrusion is complete. From the 5'-side of the extra-helical nucleoside, the helix conformation for the G- and 8-oxoG- structure varies. Consequently, when bound to G-DNA, the helix has a more pronounced bend (~80° versus ~70°); the duplex from the 5'-terminus is also over-rotated by ~20° about the vertical axis (*Fig. 7B*). This discrepancy is due to the loss of hydrogen bonds established between the 3'- and 5'-phosphates and the main chain NH-group of Asn150 and the side-chain NH-group of His270 in the complex with 8-oxoG-containing DNA. A divalent cation Ca^2+^ that coordinates the 3'-phosphate and stabilizes the bend by inner- and outer-sphere contacts to the bases flanking the extra- helical nucleoside is also missing in the G complex (*Fig. 7A*). These contacts in the 8-oxoG complex are formed only after the target base has been inserted into the lesion recognition pocket. Despite the apparent advances in understanding the structural features underlying the high specificity towards damaged base, it still remains unclear how hOGG1 recognizes 8-oxoG inside the DNA helix. According to [47] this issue could be addressed by identifying hOGG1 variants that recognize the intrahelical lesion base but with a diminished capacity for binding the lesion base outside the helix.

A structural analysis has been carried out [47] for hOGG1 variants with mutated amino acids that are in contact with 8-oxoG. As in similar studies [37], the authors employed the disulfide cross-linking strategy for irreversibly linking cysteine in hOGG1 with the C4 of cytosine complementary to the oxidized guanine. Mutations at position His270, which interacts with the 5'-phosphate (H270A mutant hOGG1), and at position Gln315 contacting with the outer face of the DNA molecule (Q315A mutant hOGG1), do not affect the structure but eliminate its functionality.

**Fig. 8:**
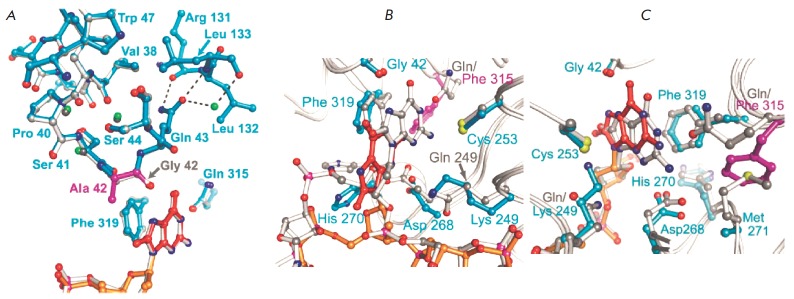
Changes in the orientation of the active site amino acids in the complex of hOGG1 with 8-oxoG-containing DNA as a result of substitutions at the amino acids involved in formation of contacts with 8-oxoG. A – Superposition of amino acid residues in the hOGG1 active sites of the WT enzyme (gray backbone) and of the G42A hOGG1 (blue backbone). B –Active site amino acids in the Q315F*149 complex; 8-oxoG residue in the WT hOGG1 is shown in gray, in Q315F hOGG1 it is shown in red. C – Active-site amino acids in the Q315F*292 complex. (Reprinted by permission from the American Society for Biochemistry and Molecular Biology: [J. Biol. Chem.] Radom C.T., Banerjee A., Verdine G.L. J. Biol. Chem. 2007. 282. 9182–9194, copyright 2007)

On the other hand, an Ala substitution at position Gly42 (G42A mutant hOGG1), removing a specific contact with 8-oxoG [36], disfavors binding of hOGG1 to DNA. As mentioned above, Gly42 is the only residue in hOGG1 that directly distinguishes between G and 8-oxoG: N7-H of 8-oxoG forms hydrogen bond with the carbonyl oxygen of Gly42 [37]. A substitution of the hydrogen atom at C_α_ of Gly42 for a bulkier methyl group of Ala sterically impedes the binding of 8-oxoG in the active site pocket (*Fig. 8A*), followed by a conformational rearrangement in hOGG1.

A mutation to Q315F, which sterically disfavored the entry of 8-oxoG into the active site pocket, was studied for two variants: Q315F*149 and Q315F*292, wherein the cytosine of complementary strand was covalently linked to Cys149 or Cys292, respectively. The Q315F*149 hOGG1 variant actually failed to insert the 8-oxoG base into the recognition pocket but only in the exo-site (*Fig. 8B*). However, Q315F*292 allowed a nearly complete insertion of 8-oxoG, thus promoting hydrogen bond formation between Gly42 and N7-H of 8-oxoG (*Fig. 8C*). The authors suggest that it is the covalent bond to the remote Cys292 residue that stabilizes the weak interaction of 8-oxoG with the recognition pocket. Notwithstanding that 8-oxoG is inserted into the pocket, no cleavage takes place. Importantly, a Gln to Phe mutation in Q315F mutant hOGG1 abrogated the specificity for both intact and 8-oxoG-DNA.

To clarify the role of certain amino acid residues in the hOGG1 active site, participating in 8-oxoG binding, a photocleavable analog of 8-oxoG carrying the C6 *o*-nitrophenylisopropyl group (PC) was synthesized [32]. The use of this analog in a DNA substrate coupled with flash-freezing shed light on the structure of a very late-stage intermediate in the base excision.

The structure of the PC-bound enzyme does not differ from that of the G-bound complex; i.e., the modified PC-base was positioned in the exo-site of the hOGG1 enzyme [37]. Upon irradiation of the crystal with 373- nm laser light for 30 s at 4°C, the PC group was cleaved, unmasking a 8-oxoG/C base pair (FC-complex). Subsequent cryotrapping in liquid nitrogen and analysis of the captured structure demonstrated that the 8-oxoG localizes in the active site pocket in the same position as in the hOGG1/8-oxoG complex [36, 47].

**Fig. 9:**
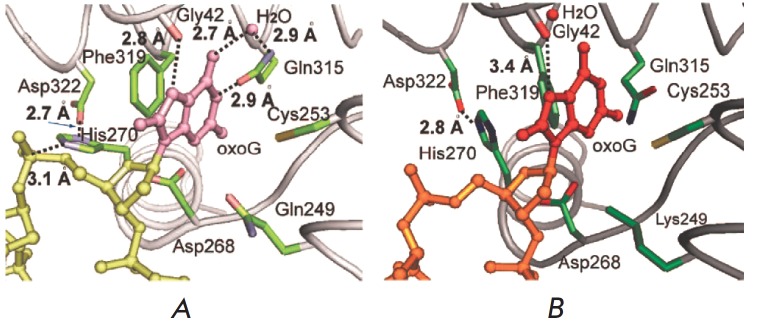
Active site view of the hOGG1/8-oxoG in the LRC complex [36] (A) and in the FC complex (B). (Reprinted by permission from American Chemical Society: [J. Am. Chem. Soc.] Lee S., Radom C.T., Verdine G.L. J. Am. Chem. Soc. 2008. 130. 7784–7785, copyright 2008)

Notably, the hydrogen bond contact , recognizing the 8-oxoG base and formed by the N7-H of 8-oxoG and the carbonyl oxygen of Gly42 is retained, although the bond is longer than that in the 8-oxoG-bound substrate (LRC-complex, 3.4 versus 2.8 A) (*Fig. 9*).

No other active site residues are in contact with 8-oxoG. The three key amino acids in hOGG1, known to establish contacts with 8-oxoG, in particular Phe319, Cys253, and Gln315, were shifted away from their positions in the FC-complex, as observed in earlier described structures [36, 47] (*Fig. 4*, *Fig. 9A*). In addition, the contact between His270 and the 5'-phosphate of 8-oxoG found in the LRC-complex is not observed in the FC-complex. This is replaced by His270 stacked with Phe319, whereas the hydrogen bond between His270 and Asp322 existing in the FC-complex is not seen in the hOGG1/8-oxoG lesion recognition complex (LRC). The catalytically important nucleophilic side-chain of Lys249 in the FC-complex is disordered and is not involved in the salt bridge with Cys253 (Lys249(NH_3_^+^)/Cys253(S^-^)) predicted to play a role in 8-oxoG recognition [37]. The difference between the FC- and LRC-complexes is not limited with side-chain motions. The α-O helix, harboring the active site residues Gln315, Phe319, and Asp322, is retracted from the active site in the FC (*Fig. 9B*).

Overall, the structure studied in [32] is the very late-stage intermediate discovered to date in the DNA glycosylase reaction, wherein the 8-oxoG base has achieved nearly complete insertion, with the active site not yet accommodated for the base excision to occur (see also [46]). It is also evidenced [32] that the transition of the target base from the exo-site to the pocket proceeds much faster than the subsequent conformational rearrangements for the active site to achieve catalytic competence.

The strategy of disulfide cross-linking [37, 47] enabled the generation of DNA-enzyme adducts in crystal form for an X-ray analysis. The hOGG1 structure, in which the intact G was extruded out of the double- stranded DNA and inserted into the pocket, was obtained and analyzed [48]. No cleavage of the N-glycosidic bond was observed. The failure to break this bond was not due to the disulfide crosslink in the enzyme– DNA adduct, since the G to 8-oxoG replacement in this complex did promote base excision. These findings indicate a mechanism by which the G base is rejected late in the base excision pathway after it has been mistakenly inserted into the hOGG1 active site. This mechanism is triggered on those rare occasions when G overcomes the transition energy barrier from the exo-site to the active site pocket. The mechanism of the rejection of G remains unknown. It was earlier shown that the N-glycosidic bond in G nucleoside is more labile to hydrolysis at neutral pH than in 8-oxoG [49]. For this reason, the discrimination between the N-glycosidic bonds of G and 8-oxoG cannot be explained by a variation in bond stability.

There is also evidence in support of the existence of a mechanism by which G is rejected once it has been presented to the recognition pocket [46]. Using stopped-flow kinetics, the catalytic effects of hOGG1 with specific and non-specific DNA substrates were studied. The combination of tryptophan and 2-aminopurine fluorescence was used to follow the conformational dynamics of DNA, as well as the conformational transitions in the enzyme. The duplex DNA molecules used contained either 8-oxoG or a non-damaged G base. When binding to hOGG1, duplex DNA exhibited double helix disruption (judged by an increase in the 2-aminopurine fluorescence) at ~10 and 20 ms, respectively. This is due to DNA bending and flipping G or 8-oxoG out of the helix [46]. At > 20 ms in the case of 8-oxoG, a decline in the 2-aminopurine fluorescence was recorded, which was likely to correspond to the entry of amino acids into the DNA gap and complex stabilization. No similar changes in the 2-aminopurine fluorescence were observed with the G-base. This means that binding of hOGG1 to a non-specific DNA substrate could lead to DNA bending and eversion of the G base into the exo-site; however, G is rejected and the enzyme fails to achieve a catalytically competent state.

Verdine *et al. *[48] proposed that the energy barrier of the transition state for breaking the N-glycosidic bond of G could be higher than that for 8-oxoG because of the conformational changes in the active site or deprivation of the transition state stabilization (enzyme-G)1 through hydrogen bonding with Gly42. In addition, the G base in the active site is in a slightly different position relative to 8-oxoG as described recently [37]. This could prevent the enzyme from adopting the optimal conformational state required for an attack on the C1' of the deoxyribose sugar by the Lys249 side-chain amino group. This is in good agreement with the finding indicating that introduction of active site mutations, that even slightly disturb 8-oxoG disposition, namely D268N [38] and Q315F [47], dramatically (but not completely) decreases the hOGG1 catalytic activity.

Lukina *et al. *[50] engineered C253L and C253I mutant hOGG1 forms with an occluded active site pocket by replacement of a Cys253 to a bulky leucine or isoleucine. Despite the perturbed active site geometry afforded by this mutation and dramatic decline in catalytic activity, the enzyme was still catalytically active. These findings argue for the concept of active site plasticity that postulates that the hOGG1 active site is flexible enough to mitigate the steric hindrance brought about by mutations [50].

The engineered complex [48] is structurally very similar to that produced with an 8-oxoG lesion. The differences in the interaction with G and 8-oxoG could be attributed to conformational adjustments to the lesion recognition pocket. It is suggested that while scanning for the target base, hOGG1 can occasionally flip the non-damaged G base into its active site [48]. The obtained data provide a basis to conclude that such erroneous insertions of G into the hOGG1 active site do not result in N-glycosidic bond cleavage due to the discrimination capacity between 8-oxoG and G at the catalytic stage.

There is a hypothesis that hOGG1 captures altered dynamic characteristics in the 8-oxoG•EC base pair relative to the G•EC base pair. This was tested by assessing the spontaneous opening of complementary pairs in double-stranded DNA using NMR spectroscopy and proton exchange [51]. It was demonstrated that the rate of spontaneous opening of 8-oxoguanine and the lifetime of the base in the extra-helical state are the same as those of a canonical guanine-cytosine base pair. This finding does not support the role of the opening dynamics of 8-oxoguanine in the recognition of the lesion by DNA glycosylases.

## CONCLUSIONS

In this review, we have focused on the structures of the DNA repair enzyme human 8-oxoguanine-DNA-glycosylase in free form and in complexes with DNA substrates. The currently available literature and data on the 3D structures of hOGG1 deposited in the Protein Data Bank (PDB) have been summarized. Lys249 and Asp268 are shown to be the key amino acids responsible for catalysis. Gly42, Asn149, Cys253, His270, Gln315, and Phe319 are the amino acids important for discrimination between 8-oxoG and G and for binding the target base in the active site pocket.

The hOGG1-mediated mechanism of oxidized base excision is reviewed in terms of structural dynamics. It obvious that the eversion of damaged base from the DNA helix into active site of enzyme cannot occur as a concerted one-step process but, it seems to proceed through multiple discrete states. It is reasonable to hypothesize that base-specific cleavage by hOGG1 is controlled throughout the interaction: lesion recognition, base extrusion, binding of 8-oxoG into the active site pocket, and the catalytic hydrolysis of the N-glycosidic bond.

## Abbreviations

AP: apurinic/apyrimidinic site
8-oxoG: 7,8-dihydro-8-oxoguanine
